# Current use and practices of lung ultrasound in geriatric care: insights from a national survey

**DOI:** 10.1007/s40520-026-03399-z

**Published:** 2026-04-17

**Authors:** Alberto Finazzi, Tessa Mazzarone, Emma Esposito, Michele Cerasuolo, Martina Salvati, Lavinia Vitali, Daniela Guarino, Nicoletta Cerundolo, Monica Torrini, Antonio Nouvenne, Fulvio Lauretani, Andrea Ungar, Giuseppe Bellelli, Raffaele Antonelli Incalzi, Dario Leosco, Andrea Ticinesi, Simone Scarlata, Chukwuma Okoye, GRETA research group contributors

**Affiliations:** 1 School of Medicine and Surgery, University of Milano-Bicocca, Milano, Italy; 2 Geriatrics Unit, Department of Clinical and Experimental Medicine, University of Pisa, Pisa, Italy; 3 Department of Advanced Medical and Surgical Sciences, University of Campania “Luigi Vanvitelli”, Naples, Italy; 4 Department of Medicine and Surgery, Research Unit of Geriatrics, Campus Bio-Medico University, Rome, Italy; 5 Department of Care Continuity and Multicomplexity, Azienda Ospedaliero- Universitaria di Parma, Parma, Italy; 6 Geriatrics and Intensive Care Unit, Azienda Ospedaliero-Universitaria Careggi, Florence, Italy; 7 Department of Medicine and Surgery, University of Parma, Parma, Italy; 8 Department of Experimental and Clinical Medicine, University of Florence, Florence, Italy; 9 Department of Translational Medical Sciences, University of Naples Federico II, Naples, Italy; 10 Department of Medical Sciences, University of Ferrara, Via Luigi Borsari 46, Ferrara, 44121 Italy

**Keywords:** Lung ultrasound, Geriatric acute care, Point-of-care ultrasound, Survey, ultrasound training

## Abstract

**Background:**

Lung ultrasound (LUS) has gained increasing relevance in the evaluation of respiratory symptoms due to its bedside applicability, diagnostic accuracy, and safety. However, its adoption in geriatric care remains limited.

**Aims:**

This study aimed to assess current LUS availability, use, and technical practices among Italian geriatricians.

**Methods:**

A nationwide, cross-sectional survey was conducted among members of the Italian Society of Gerontology and Geriatrics. The 44-item questionnaire explored LUS availability, use, indications, and technical practices in acute care. LUS proficiency was operationally defined as the ability to perform the scan, interpret findings and integrate them with clinical data independently or under supervision.

**Results:**

Responders (*n* = 154), representing 57 hospitals in 17 Italian regions, reported wide interest in LUS and recognized its utility for improving the care of older patients with acute respiratory failure. The main indications, which LUS was used for in their clinical experience, were assessment of volume status, pleural effusions and heart failure. Although availability of LUS equipment in the clinical units of responders was generally high (94%), 27.3% of them were non-proficient in LUS and more than 85% reported that a trained operator was not available 24/7. Heterogeneity also emerged in examination techniques and reporting among LUS-proficient responders, with only 6% adopting validated scanning protocols and 13% using standardized reporting formats.

**Conclusion:**

This survey reveals limited integration of LUS in geriatric care with heterogeneous practices. Structured training pathways and geriatric-specific standardized LUS protocols are urgently needed to enable broader, safer, and more consistent implementation in acute geriatric care.

**Supplementary Information:**

The online version contains supplementary material available at 10.1007/s40520-026-03399-z.

## Introduction

Lung ultrasound (LUS) has emerged as a valuable bedside tool for the assessment of respiratory conditions, extending beyond its original use in emergency and intensive care settings [1, 2].

Growing evidence supports its promising role in geriatric medicine [3, 4]. When integrated with clinical and laboratory evaluation, LUS has demonstrated greater diagnostic accuracy than chest radiography (CXR) in detecting pneumonia, heart failure (HF), pleural effusion, and acute exacerbations of chronic obstructive pulmonary disease (COPD) [5–8]. Its value is further enhanced by age-related anatomical changes that may reduce the accuracy of CXR and chest computed tomography (CT) [9]. Additionally, the diagnostic yield of LUS is preserved in older adults with restricted mobility or cognitive impairment, which often hinder the use of other imaging procedures. Finally, it is a cost-effective tool that allows frequent bedside reassessment, especially valuable in managing complex geriatric patients [4].

Despite its well-established benefits, LUS remains underused in geriatric settings. LUS, in fact, was originally developed mainly in the Emergency Department (ED) and Intensive Care Unit (ICU) setting, where it rapidly spread for its enormous advantages in terms of rapidity of execution and diagnostic accuracy in critical situations. In the last twenty years, LUS gradually spread also to internal medicine wards dealing with acute respiratory patients establishing, in some cases, a novel standard of care. However, the popularity of point-of-care ultrasound in general, and LUS in particular, is still limited among geriatricians. Standardized approaches specifically tailored to the needs of older patients are currently unavailable, and the evidence base in this population is still lacking [3]. Furthermore, only a small number of studies have specifically investigated its application in older patients.

Preliminary insights were offered by a prior survey among Italian geriatric wards, which outlined early patterns of use and signalled relevant unmet needs in clinical practice [10]. However, this survey was addressed to department heads and covered only general issues, thereby excluding the direct perspectives of clinicians and trainees actively engaged in daily ultrasound practice. Furthermore, the survey predated the COVID-19 pandemic, a period that profoundly reshaped diagnostic workflows and accelerated the uptake of point-of-care ultrasound across acute care settings. Importantly, the survey did not address key technical aspects of LUS implementation, such as examination protocols and reporting practices.

To address these gaps, we conducted a nationwide survey targeting Italian geriatricians, with the aim of providing an updated and practice-oriented overview of LUS utilization in geriatric care. The survey explored three core domains: (1) the current level of adoption and integration of LUS within geriatric services; (2) clinicians’ perceptions of its clinical relevance, particularly in the assessment and management of acute respiratory failure (ARF); and (3) the technical modalities underpinning its use, including examination protocols, and reporting procedures.

## Methods

### Study design and data collection

A cross-sectional survey was conducted in Italy between July 2023 and July 2024. The survey targeted physicians who are active members of the Italian Society of Geriatrics and Gerontology (*Società Italiana di Geriatria e Gerontologia*, SIGG). The questionnaire was developed by the Research Group on Thoracic Ultrasound in Older Patients (*Gruppo di Ricerca sull’Ecografia Toracica nell’Anziano*, GRETA), a SIGG-affiliated group established in 2018 to promote awareness, research, and clinical integration of LUS in geriatric care through education and scientific collaboration. To assess clarity and feasibility, the questionnaire was pre-tested among a small group of geriatricians collaborating with GRETA Group members, who provided positive feedback.

The survey was disseminated via email through the active SIGG members’ mailing list over a one-year period. All registered members were invited to participate, and up to four reminder emails were sent to non-respondents to maximize participation. The total number of physicians who were reached through the SIGG mailing list was 1412. Among them, 35% were young geriatricians or residents (having obtained their MD degree less than five years before). The geographical distribution of recipients was homogeneous across Northern, Central and Southern Italy. Recipients were also encouraged to forward the invitation to colleagues to broaden dissemination. The survey was open and anonymous.

The questionnaire consisted of 44 questions assessing the spread of LUS utilization across different Italian regions and hospital wards and the current LUS practices (**Supplementary Material 1**). The main domains explored were LUS availability, indications, training, technical protocols, and reporting strategies. The geographical provenience of respondents was categorized into three groups: Northern Italy (Valle d’Aosta, Piemonte, Liguria, Lombardia, Trentino-Alto Adige, Veneto, Friuli-Venezia Giulia, Emilia-Romagna), Central Italy (Toscana, Umbria, Marche, Lazio) and Southern Italy (Abruzzo, Molise, Campania, Puglia, Basilicata, Calabria, Sicilia, Sardegna). The questionnaire also explored the characteristics of LUS operators, including their professional roles and years of experience.

Prior to dissemination, the questionnaire underwent structured pre-testing involving 12 senior geriatricians with established experience in lung ultrasound and acute geriatric care. Participants were asked to evaluate item clarity, relevance, completeness, and clinical appropriateness. Minor wording refinements were implemented accordingly. This process aimed to ensure face and content validity. Formal psychometric testing, including test–retest reliability assessment, was not performed, as the survey was designed as an exploratory descriptive instrument rather than a scale-based measurement tool.

Respondents were classified as LUS proficient if able to perform examinations, interpret findings and integrate them with clinical and laboratory data independently or under supervision, while as non-proficient if unable. The survey also examined indications for LUS use among both groups and its role in the diagnostic approach for patients presenting with ARF.

### Statistical analysis

Socio-demographic and participants’ responses were analysed using descriptive statistics. Categorical variables were summarized as absolute frequencies and percentages, while continuous variables were expressed as medians with interquartile ranges (IQR). Pearson’s chi-square test was used to compare categorical variables. To evaluate the different perspective among LUS proficient and LUS non-proficient about LUS indications in geriatric clinical practice, a dot plot was generated showing the corresponding p-values. To further explore technical aspects of LUS, such as methodology, imaging approach, and reporting techniques, a sensitivity analysis was conducted among LUS proficient only. Data were collected using an electronic case report form (eCRF) by trained personnel via Research Electronic Data Capture (REDCap) [11]. Analyses were conducted using R software, version 2024.04.227 [12].

## Results

### Characteristics of study respondents and adoption of LUS in Italian geriatric units

A total of 154 physicians, representing 57 hospitals across seventeen Italian regions, responded to the questionnaire, with a prevalence of responders from Northern Italy and from geriatricians or geriatricians in training working in university hospitals **(**Table 1). The response rate, calculated considering physicians contacted through the SIGG mailing list, was 10.9%.

**Table 1:** Survey characteristics and LUS diffusion

	*n* = 154
Macro-area, *n* (%)	
Northern Italy	92 (59.7)
Central Italy	32 (20.8)
Southern Italy	30 (19.5)
University hospital setting, n (%)	115 (74.7)
Professional qualification, n (%)	
Trained Geriatricians	56 (36.6)
Geriatrics residents	98 (63.4)
Availability of ultrasound machine, n (%)	145 (94.2)
LUS proficiency, n (%)	
Yes, autonomously	53 (34.4)
Yes, under supervision	59 (38.3)
No	42 (27.3)
Number of trained geriatricians performing LUS in the ward, median (IQR)	3 (2–5)
Number of bedside LUS executed weekly in the ward, n (%)	
Less than 10	97 (63)
Between 10 and 20	42 (27.3)
More than 20	15 (9.7)

**Fig. 1:**
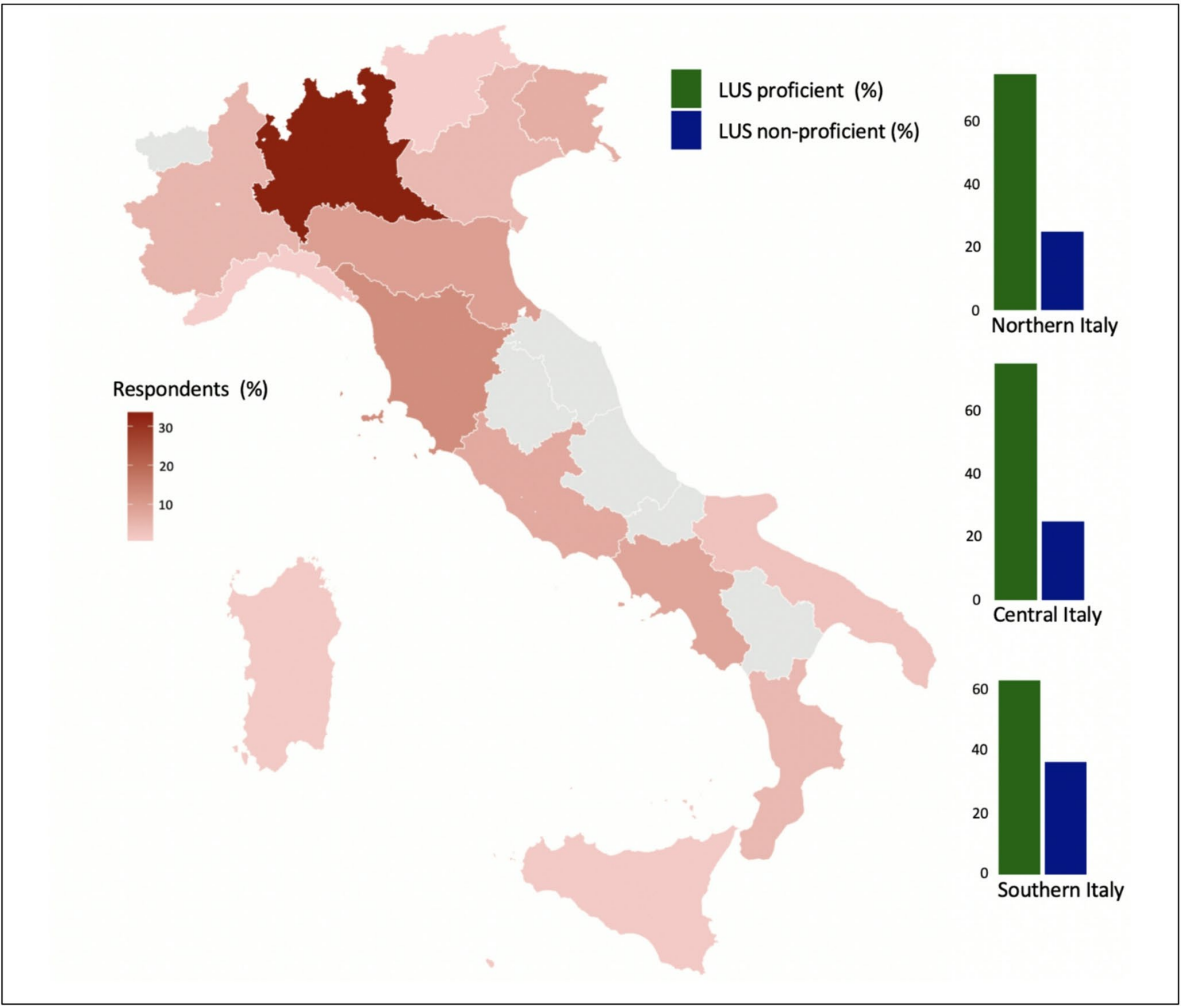
Heat map of Italy showing the percentage of respondents by region. The bar charts represent the percentage of LUS proficient (green) vs. LUS non-proficient (blue) divided by macro-area (Northern, Central, and Southern Italy)

Nearly all participants had access to at least one ultrasound equipment in their ward (94.2%), most commonly a conventional portable system with convex and linear probes (> 75%), and less frequently a handheld device connectable to tablets or smartphones (4.2%) or both. Overall, 72.7% of respondents were LUS proficient, whereas 27.3% were LUS non-proficient, while, even if proficient, only 34.4% of the whole sample were able to perform LUS autonomously (Table 1). Most participants (53.9%) learned the method from more experienced colleagues, while 38.5% reported having acquired it through courses organized by scientific societies. Finally, 7.7% reported being self-taught. As shown in Fig. 1, LUS proficiency was numerically lower in Southern Italy compared with Northern and Central Italy; however, these differences were not statistically significant.

Respondents indicated a median of three geriatricians (IQR, 2–5) proficient in LUS in their wards, but in most cases the number of bedside LUS examinations performed weekly was lower than 10 (Table 1). In fact, more than 85% of respondents reported that a trained operator was not available 24/7 for performing LUS in their ward.

### LUS for the diagnosis and management of ARF

The survey explored the practices reported by all respondents, both LUS-proficient and non-proficient, on the use of LUS in managing ARF in older patients according to their clinical experience. As shown in Table 2, most respondents indicated that LUS was the preferred initial imaging modality for evaluating ARF in their emergency department in only 19.5% of cases, while standard approach with CXR prescription as first imaging test remained widespread. Most respondents estimated that fewer than 25% of older patients admitted to their units with ARF were assessed with LUS (Table 2). LUS was in fact frequently used electively or for clinical monitoring. Only 39.6% of respondents reported that LUS was routinely performed in their wards at the time of admission, alongside physical examination or within the first 24 h of hospitalization. In further 6.5% of cases, LUS was reported to be performed exclusively in emergencies or in case of clinical deterioration. Finally, 9.1% indicated that LUS was not employed at all for ARF assessment in their wards. When used, LUS was performed independently of CXR findings according to most respondents, while it was considered as a substitute for CXR in only a small number of cases (Table 2).

**Table 2:** Application of LUS in older patients with acute respiratory failure

	*n* = 154
First-Line Diagnostic Methodic, *n* (%)^†^	
Chest X-Ray (CXR)	117 (76)
LUS	30 (19.5)
Chest CT	5 (3.2)
LUS execution in patients hospitalized for ARF, n (%)^‡^	
Less than 25%	66 (42.9)
Between 25 and 50%	43 (27.9)
Between 50 and 75%	28 (18.2)
More than 75%	14 (9.0)
Timing of LUS use, n (%)^*^	
At Admission with Physical Examination (≤ 24 h)	61 (39.6)
Electively (Machine/Sonographer Available) or Clinical Monitoring	68 (44.2)
For Clinical Worsening	10 (6.5)
Not performed in ARF	14 (9.1)
LUS employment compared to CXR in ARF, n (%)	
Independently of CXR findings	91 (59.0)
Adjunctive Use for Unclear CXR	34 (22.1)
As substitute for CXR	9 (5.8)
Others	25 (16.2)
LUS utility in diagnostic approach of ARF, n (%)^†^	
Always	110 (71.4)
Only when CXR is negative	9 (5.8)
Only in specific situations	33 (21.4)
Never	0 (0)
Frequency of LUS influence in clinical management, n (%)^†^	
Often	65 (42.22)
Only in some cases	59 (38.3)
Rarely	22 (14.2)
Never	6 (3.9)
Opinion on reduction of CT prescription giving the LUS employment, n (%)	57 (37)

Abbreviations: ARF: acute respiratory failure; CT: computed tomography: N/A: non available

* 1 missing value (0.6%)

† 2 missing values (1.3%)

‡ 3 missing values (1.9%)

Around 70% of respondents believed that LUS use improved or would improve the diagnostic approach for ARF in their wards, in comparison with CXR use only. Others either considered LUS useful only in specific situations (21.4%) or found it beneficial only when CXR findings are negative (5.8%). All responders disagreed with the statement that LUS is not useful in the assessment of acute respiratory conditions. Additionally, 42.2% of participants stated that LUS frequently modified clinical management of patients, and 37% reported that its use contributed to a reduction in chest CT prescriptions, according to their personal experience.

### LUS application in geriatric clinical practice and differences according to LUS proficient and LUS non-proficient

**Fig. 2:**
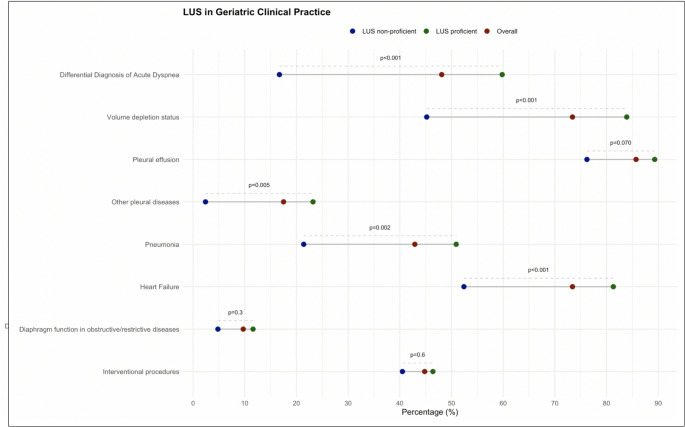
Respondents’ opinions on LUS indications for the diagnosis and monitoring of selected respiratory and cardiovascular conditions, by proficiency level (all respondents = red, LUS-proficient = green, LUS non-proficient = blue)

As shown in Fig. 2, respondents reported multiple perceived clinical uses for LUS in geriatric practice. The most frequent uses reported by responders according to their experience were detection of pleural effusions (85.7%), assessment of volume status (73.4%), and evaluation of HF (73.4%). Nearly half of respondents reported that LUS was mainly used for the differential diagnosis of acute dyspnea (48.1%), for the diagnosis of pneumonia (42.9%), and for guiding interventional procedures (44.8%). When comparing proficient with non-proficient operators, significant differences were observed across most reported clinical indications. LUS proficient were more prone to consider LUS as indicated for the differential diagnosis of acute dyspnea (59.8% vs. 16.7%; *p* < 0.001), for the assessment of volume status (83.9% vs. 45.2%; *p* < 0.001), for the diagnosis of pneumonia (50.9% vs. 21.4%; *p* = 0.002), and for evaluation of HF (81.3% vs. 52.4%; *p* < 0.001). No significant differences emerged between proficient and non-proficient responders’ perceived LUS clinical uses regarding detection of pleural effusion (89.3% vs. 76.2%; *p* = 0.070), diaphragm function assessment in obstructive or restrictive diseases (11.6% vs. 4.8%; *p* = 0.30), and guidance of interventional procedures (46.4% vs. 40.5%; *p* = 0.60).

### Current practices in LUS application among LUS proficient: setting, technique, and reporting

Among the 112 LUS proficient responders, reporting a median of 4 years of experience (IQR, 2.0–6.5), more than 90% performed LUS at the bedside, whereas only 16.1% used a dedicated room (**Supplementary Table 1**). The techniques of LUS execution were very heterogeneous, with only 6.3% of proficient operators reporting the use of a validated protocol despite the availability of ultrasound equipment. In a significant number of LUS proficient responders (31.3%), the technique changed according to different clinical indications (**Supplementary Table 1**). The reporting method of LUS findings was also heterogeneous among LUS proficient responders. In 56.3% of cases no specific reporting method was used, while 31.3% mentioned the use of a reporting approach shared among ultrasound specialists. Only 12.6% of LUS proficient responders reported the use of a standardized reporting protocol, which was scientifically validated in the literature in even fewer cases (**Supplementary Table 1**).

The survey further investigated the reporting protocols for typical ultrasound patterns of acute HF and pulmonary edema, specifically the quantification of interstitial congestion and pleural effusions. Regarding the severity of interstitial involvement, most responders (59.8%) reported the quantification of B-lines as an indirect measure of severity, while only 7.1% employed a validated tool such as the LUS score [13]. In 33% of cases, no method to describe severity of lung parenchyma interstitial involvement was used. In the evaluation of pleural effusion, nearly 60% of respondents reported the count of intercostal spaces where the effusion is visible as a measure of severity. In 24.1% of cases effusion severity was estimated through the measurement of the maximum thickness, while only 4.5% of responders declared the use of validated formulas to quantify the effusion volume.

Finally, diaphragmatic ultrasound was not common among geriatricians, with only 12.5% of responders reporting its use. In all these cases, the assessment included diaphragmatic excursion, while 57.1% also measured changes in diaphragmatic thickness and 21.4% evaluated the length of the diaphragmatic zone of apposition (**Supplementary Table 1**).

## Discussion

In this nationwide survey of Italian geriatricians, we found that LUS is widely available but not consistently incorporated into routine geriatric practice. Although almost all respondents reported having access to an ultrasound device, only around 70% can be considered as LUS proficient in terms of capacity to perform examinations, interpret findings and integrate them with clinical and laboratory data. More than 85% indicated that a trained operator was not routinely available in their ward to perform LUS daily. Notably, LUS was perceived as a valuable diagnostic and management tool for ARF, especially among those familiar with its use, underscoring the importance of expanding its adoption. Finally, our findings highlight substantial heterogeneity in technical approaches and reporting methods, emphasizing the need for the adoption of standardized protocols in geriatric care.

Although LUS is gaining ground in geriatric settings, the limited number of operators able to perform examinations autonomously and the limited availability of LUS proficient physicians in wards suggest that its implementation remains incomplete. The findings on ARF reinforce this, showing that, while most respondents regard LUS as a valuable tool—often used independently of, or alongside, CXR—its routine application in the early diagnostic phase is uncommon. Instead, LUS is frequently reserved for clinical monitoring or when CXR findings are inconclusive, suggesting that its potential to expedite diagnosis and reduce reliance on chest CT is not fully realized in geriatric practice yet. Previous surveys, including the one by the Italian Academy of Thoracic Ultrasound (*Accademia di Ecografia Toracica*) among Italian pulmonologists [14], identified multiple barriers to LUS implementation such as limited equipment availability, lack of time for performing examinations, limited training, and perception that LUS mainly belongs to the expertise of other specialists. Although our survey did not explore perceived barriers, these factors may also be relevant to our setting.

In line with our previous survey conducted in geriatric units [10], our results revealed no significant lack of equipment, as 90% of respondents reported access to an ultrasound machine within their facility. Regarding training, the majority had developed LUS skills either through self-directed learning or by receiving informal instruction from more experienced colleagues, while only 38.5% had benefited from structured, course-based training. This confirms the limited availability of structured ultrasound training for geriatricians, as previously noted in other surveys [10, 15], and highlights regional disparities in access to such programs. The circumstance that most responders to our survey either worked in academic hospitals or were geriatricians in training further underscores the key role of academic institutions in promoting LUS use among young physicians, in line with current guideline recommendations [16–19]. Finally, while our survey did not address the perception of LUS as a technique predominantly employed by other specialists, its origins in emergency and intensive care settings likely contribute to its more limited adoption in geriatric practice. Nevertheless, previous geriatric surveys reveal strong interest among geriatricians in developing bedside ultrasound skills [20].

The confidence in LUS among geriatricians may be influenced by challenges in interpreting certain findings, such as B-lines, whose presence spans multiple pulmonary conditions, particularly in older adults with multimorbidity and multiple overlapping causes of dyspnea. These artifacts, indicative of diffuse interstitial involvement and impaired alveolar–capillary exchange, can occur in a wide range of conditions, including cardiogenic and non-cardiogenic pulmonary oedema, primary or secondary pulmonary fibrosis, and various types of infection [8, 21]. The interpretation of these artifacts may be also intrinsically challenging and prone to misclassification, because of multiple cardio-respiratory conditions overlapping in older individuals and intrinsic modifications of the anatomy of the respiratory system occurring with aging. Nevertheless, the adoption of established ultrasound protocols, like the BLUE protocol demonstrating high diagnostic accuracy in ARF [22], and current guidelines endorsing LUS as complementary or alternative to CXR [2, 7, 16, 23–26]—particularly in patients with reduced mobility or requiring serial assessments— highlight the considerable potential of LUS. Notably, 42.2% of physicians in our survey reported LUS-driven changes in patient management, including more targeted treatments and avoidance of unnecessary CT scans, further underscoring its value as a dynamic bedside tool for optimising care pathways in older adults with ARF [3].

The recognition of the importance of LUS in different specific respiratory conditions, that was particularly evident in LUS-proficient responders, further confirms its potential in geriatric care. Notably, LUS was perceived as a highly valuable diagnostic and management tool, particularly in the assessment of pleural effusion, volume status, and HF. All these applications are supported by robust evidence and well-established protocols [16]. The greater confidence expressed by LUS-proficient responders is probably related to their deeper understanding of the technique’s potential and its diagnostic accuracy relative to conventional radiology. This attitude is consistent with existing literature demonstrating the quantitative and qualitative utility of LUS in pleural effusion [27], its role in the stratification and monitoring of HF [28], and its reliability in pneumonia diagnosis [29]. However, a certain degree of familiarity bias and confirmation bias cannot be excluded, as those already skilled in the method may be more prone to perceive it positively. Recognizing this possibility underscores the importance of structured training programs and objective comparative evaluations to promote broader adoption of LUS in geriatric care [4].

Another challenge emerging from our survey is the lack of standardisation in LUS examination and reporting, with marked heterogeneity in methods of examination, such as the number of lung fields assessed, and documentation, particularly concerning the quantification of B-lines and pleural effusions. These inconsistencies reflect the broader absence of protocols specifically tailored to older adults. Developing shared standards, covering technical parameters, interpretation criteria and reporting formats could improve reproducibility, diagnostic accuracy, and longitudinal monitoring of respiratory conditions, ultimately enhancing decision-making and outcome prediction in geriatric care. Consensus recommendations propose a 12-zone scanning system as an optimal compromise between feasibility and diagnostic yield, alongside semi-quantitative grading of B-lines to improve consistency and clinical interpretability [30]. The use of validated formulas to estimate pleural effusion volume is preferred, though a semi-quantitative classification (minimal, moderate, extensive, based on the number of intercostal spaces involved) remains a practical alternative when calculation is not feasible. These measures support reproducibility, facilitate follow-up, and enable longitudinal assessment. Overall, the reliance on protocols not specifically tailored to older patients likely contributes to the current lack of standardization. Scientific societies should therefore develop dedicated guidelines for older patients treated in geriatric settings, specifying a minimum set of lung areas to assess, validated semi-quantitative metrics for interstitial congestion and pleural effusion, standardized report formats compatible with electronic medical records, and uniform competency certification to ensure consistent training nationwide [4]. The GRETA group, endorsed by SIGG, aims to address this gap through a comprehensive review of the topic, the design of a national multicenter study on LUS in older adults [31], and the ongoing organization of national and regional training courses.

### Limitations and strengths

Although this survey provides a timely and valuable overview of LUS use in geriatric settings, several methodological limitations constrain the generalisability of its findings and warrant cautious interpretation. First, the sample was self-selected and composed of physicians affiliated with the SIGG, a scientific society that actively promotes LUS training. The online sampling technique and the dissemination strategy (email invitations sent to personal addresses, with multiple reminders to non-respondents) may have introduced selection bias. Furthermore, the calculated response rate (10.9%) represents an estimation based on the number of physicians originally contacted through the SIGG mailing list and not on actual recipients. The survey was also addressed to individual physicians and did not collect information on the specific geriatric ward within each hospital. Thus, multiple respondents may have come from the same institution, potentially introducing some duplication in responses to organizational questions. However, given the large number of hospitals represented, this risk is likely limited.

Moreover, most participants were based in academic institutions, where access to equipment, experienced tutors, and structured educational programmes is typically greater. A considerable proportion of respondents were geriatric residents, who are likely to have increased awareness of the potential of LUS in clinical practice. These circumstances may limit the generalizability of results to non-academic institutions. Objective outcome measures were not considered in this study, which only explored the impact of LUS implementation perceived by responders. The definition of LUS proficiency was also based on self-reported evaluations of responders, and not on objective assessment or certified numbers of previous examinations. Thus, our data cannot support any causal inference on the real clinical impact of LUS. Finally, although the questionnaire was reviewed by experienced geriatricians to ensure clarity and content relevance, it did not undergo formal psychometric validation. As such, its measurement properties were not formally assessed.

Taken together, these factors may have led to an overestimation of LUS availability, utility, and expertise nationwide, but they should also be viewed as a motivation to further expand and strengthen LUS training across the country. Secondly, respondents were predominantly from Northern Italy, with fewer from Central and Southern regions, which may limit geographical representativeness of the survey, especially considering that the distribution of physicians affiliated with SIGG is homogeneous across the country. This imbalance may reflect regional inequalities in LUS use and methodological expertise.

## Conclusions

This nationwide survey shows that LUS remains only partially integrated into geriatric practice, with few trained operators and considerable variability in its application and reporting. Although respondents recognised its value, particularly in ARF, pleural effusion, volume status assessment, and HF, its full potential is limited by training gaps, interpretative challenges, and the absence of geriatric-specific protocols. Given its promising relevance in geriatric care, greater efforts are needed to enhance technical expertise and promote standardisation of LUS in this setting.

## Supplementary Information

Below is the link to the electronic supplementary material.

Supplementary Material 1

Supplementary Material 2

## Data Availability

Raw data on the survey on LUS are available upon reasonable request to the corresponding author.
