# Supplementary material for: Current use and practices of lung ultrasound in geriatric care: insights from a national survey
Source: Aging Clin Exp Res. 2026 Apr 17;38(1):141. doi: 10.1007/s40520-026-03399-z (PMC13216130; doi:10.1007/s40520-026-03399-z)
Supplement: Supplementary file 1 — Supplementary Material 1 [file 40520_2026_3399_MOESM1_ESM.docx]

**SURVEY ON THE USE OF LUNG ULTRASOUND IN GERIATRIC UNITS IN ITALY**

**Unit and Hospital Characteristics**

**Hospital name**

**University hospital**

□ Yes

□ No

**Is the unit under university leadership?**

□ Yes

□ No

**In which Italian region is your hospital located?**

□ Valle d’Aosta

□ Piemonte

□ Liguria

□ Lombardia

□ Trentino-Alto Adige/Südtirol

□ Veneto

□ Friuli-Venezia Giulia

□ Emilia-Romagna

□ Toscana

□ Umbria

□ Marche

□ Lazio

□ Abruzzo

□ Molise

□ Campania

□ Puglia

□ Basilicata

□ Calabria

□ Sicilia

□ Sardegna

**Number of beds in the unit ____**

**Professional Profile**

**What is your professional role?**

□ Specialist physician

□ Resident physician

**Years of experience as a specialist**

□ Less than 5 years

□ 5–10 years

□ More than 10 years

**Availability and Use of Ultrasound**

**Is an ultrasound machine available in your unit to perform bedside diagnostics?**

□ Yes

□ No

If yes, **which types of ultrasound devices are available?**

□ Standard portable ultrasound machines with convex and linear probes

□ Class II portable ultrasound systems with probes connected to tablets or smartphones

□ Both

**Is bedside internal medicine ultrasound (abdomen, vascular, thyroid, FAST exam) performed in your unit?**

□ Yes

□ No

If yes, **how often?**

□ Routinely in all patients with clinical indication

□ Only in selected cases based on clinical presentation

□ Only depending on equipment availability

□ Only depending on the availability of a trained physician

**Which ultrasound examination is most commonly performed at the bedside in your ward?**

□ Cardiac ultrasound

□ Abdominal ultrasound

□ Lung ultrasound

□ Vascular ultrasound

**Number of attending physicians able to perform lung ultrasound**

**Are residents able to perform lung ultrasound under supervision?**

□ Yes

□ No

**Is there a physician available to perform lung ultrasound bedside 24/7?**

□ Yes

□ No

**Approximate number of bedside lung ultrasounds performed weekly in your unit**

□ <10

□ 10–20

□ 20–30

□ 30

**Clinical Settings of Use**

**In which clinical settings is lung ultrasound used in your unit? (multiple answers possible)**

□ Intensive or sub-intensive care

□ Acute care ward

□ Long-term care / chronic patients

□ Outpatient clinic

□ Community setting (e.g., nursing homes, home hospital services)

**Where is lung ultrasound performed in hospitalized patients?**

□ At the bedside

□ In a dedicated ultrasound room within the ward

□ In an ultrasound room outside the ward

□ Not applicable

**Technical Approach**

In patients able to sit upright, **how is lung ultrasound performed in your unit?**

□ Only posterior lung regions are scanned

□ Posterior and lateral regions are scanned

□ Posterior, lateral, and anterior regions are scanned

□ A standardized protocol is applied (e.g., Soldati protocol with Lung Ultrasound Score)

□ No standardized approach; technique varies depending on patient and clinical question

**Clinical Indications**

**For which indications is lung ultrasound used in your unit? (multiple answers possible)**

□ Differential diagnosis of acute dyspnea

□ Assessment of volume status in acute patients (fluid overload vs depletion)

□ Diagnosis and follow-up of pleural effusion

□ Diagnosis and follow-up of other pleural diseases

□ Diagnosis and follow-up of pneumonia

□ Diagnosis and follow-up of heart failure

□ Evaluation of diaphragmatic function in chronic restrictive or obstructive diseases

□ Clinical monitoring of diseases diagnosed with other imaging methods

□ Interventional procedures (thoracentesis, central venous catheter placement)

**Imaging Strategy in Acute Respiratory Failure**

In an older patient admitted from the emergency department with **acute dyspnea or respiratory failure**, which imaging test is usually performed first?

□ Chest X-ray

□ Bedside lung ultrasound

□ HRCT / contrast-enhanced chest CT

**Approximate percentage of hospitalized patients with acute respiratory failure who receive bedside lung ultrasound during admission**

□ <25%

□ 25–50%

□ 50–75%

□ 75%

**Timing of bedside lung ultrasound in these patients**

□ Immediately at admission together with physical examination

□ Within 24 hours of admission

□ Electively depending on equipment and operator availability

□ Only in case of clinical deterioration

□ Only for clinical monitoring

□ Not used in acute patients

**Relationship Between Lung Ultrasound and Chest X-ray**

In acute patients, lung ultrasound is used:

□ As a replacement for chest X-ray

□ As a complement to chest X-ray regardless of results

□ As a complement only when chest X-ray is negative or inconclusive

□ Other

**Pneumonia Evaluation**

When patients develop **fever during hospitalization** with high suspicion of pulmonary infection, which imaging test is usually performed first?

□ Chest X-ray

□ Bedside lung ultrasound

□ HRCT / chest CT with contrast

If lung ultrasound is performed first and **pneumonia is suspected**, what is the next step?

□ No further imaging; therapy is started based on clinical and microbiological findings

□ Chest X-ray confirmation

□ Chest CT confirmation if clinically indicated

If ultrasound findings **are not clearly suggestive of pneumonia**, what is the next step?

□ Clinical monitoring without further imaging

□ Chest X-ray

□ Chest CT

**Quantification of Pulmonary Congestion**

In patients with **heart failure or acute pulmonary edema**, how is the severity of interstitial involvement estimated?

□ Counting B-lines per scanning field

□ Lung Ultrasound Score (LUS)

□ No severity assessment performed

□ Other (specify)

**Pleural Effusion Assessment**

How is pleural effusion severity estimated?

□ Counting the number of intercostal spaces involved

□ Measuring maximal effusion thickness

□ Using validated formulas to estimate effusion volume

□ Other (specify)

**COVID-19**

Since 2020, **have COVID-19 patients been treated in your unit?**

□ Yes

□ No

If yes, how has lung ultrasound been used?

□ Diagnosis of interstitial pneumonia in all patients

□ Diagnosis only when chest X-ray/CT was non-diagnostic

□ Monitoring evolution of lung lesions

□ Guiding escalation of oxygen or ventilatory support

□ Evaluating discharge readiness

□ Monitoring post-COVID or long-COVID lung sequelae

**Integration With Other Ultrasound Examinations**

Is lung ultrasound **integrated with bedside echocardiography?**

□ Yes

□ No

If yes, who performs echocardiography?

□ Same physician at the same time

□ Same physician at different times

□ Another physician at similar times

□ Another physician at separate times

Is lung ultrasound integrated with **diaphragm ultrasound?**

□ Yes

□ No

If yes, which measurements are used?

□ Diaphragmatic excursion

□ Diaphragm thickness variation

□ Length of diaphragmatic zone of apposition

In which patients is diaphragm ultrasound performed?

□ Mechanically ventilated patients (weaning assessment)

□ Restrictive or neuromuscular diseases

□ Obstructive diseases/COPD

□ Acute respiratory failure

□ Other

**Operator Experience**

Are you able to perform bedside lung ultrasound?

□ Yes, independently

□ Yes, under supervision

□ No

If yes, **how many years of experience do you have?**

Which level best describes your expertise?

□ **Basic level** (pleural effusion, volume status, gross parenchymal abnormalities)

□ **Intermediate level** (differential diagnosis of acute respiratory failure, pleural diseases, parenchymal consolidations)

□ **Advanced level** (diaphragm ultrasound, integration with echocardiography)

Where did you learn lung ultrasound?

□ Self-taught

□ From colleagues during the COVID-19 pandemic

□ From colleagues before the pandemic

□ Scientific society courses

□ GRETA-SIGG course

**Reporting and Clinical Impact**

Is there a **standardized reporting system** for lung ultrasound in your unit?

□ Yes, validated tool from literature

□ Yes, internal tool

□ No, but shared reporting practices exist

□ No, each physician reports independently

Based on your experience, **does lung ultrasound improve diagnostic accuracy compared with chest X-ray in patients with dyspnea or respiratory symptoms?**

□ Always

□ Only when chest X-ray is negative

□ Only in specific situations

□ Never

How often do lung ultrasound results **change clinical management**?

□ Very often

□ Sometimes

□ Rarely

□ Never

Do you think lung ultrasound has **reduced the number of chest CT scans** requested in your unit?

□ Yes

□ No

□ Not sure
