# Supplementary material for: Current use and practices of lung ultrasound in geriatric care: insights from a national survey
Source: Aging Clin Exp Res. 2026 Apr 17;38(1):141. doi: 10.1007/s40520-026-03399-z (PMC13216130; doi:10.1007/s40520-026-03399-z)
Supplement: Supplementary file 2 — Supplementary Material 2 [file 40520_2026_3399_MOESM2_ESM.docx]

**SUPPLEMENTARY MATERIAL 2**

**Supplementary Table 1.** Sensitivity analysis on LUS proficient.

|  | n = 112 |
| --- | --- |
| Years of LUS Experience, median (IQR) | 4 (2.0−6.5) |
| Bedside LUS application, n (%) | 105 (93.8) |
| Dedicated room, n (%) | 18 (16.1) |
| LUS execution technique, n (%) |  |
| Only posterior | 10 (8.9) |
| Posterior and lateral | 18 (16.1) |
| Posterior, lateral, and anterior | 42 (37.5) |
| Validated protocol | 7 (6.3) |
| According to clinical question | 35 (31.3) |
| Reporting of LUS findings in your department, n (%) |  |
| Yes, Literature-Based Protocol | 7 (6.3) |
| Yes, Internal Protocol | 7 (6.3) |
| No, Shared Reporting Approach | 35 (31.3) |
| No Protocol, No Shared Reporting Approach | 63 (56.3) |
| Measure of interstitial involvement severity in heart failure or acute pulmonary edema, n (%) |  |
| Description and quantification of B-lines | 67 (59.8) |
| Use of lung ultrasound score (LUS score) | 8 (7.1) |
| None | 37 (33.0) |
| Measure of pleural effusion severity, n (%) |  |
| Quantification of intercostal spaces interested | 67 (59.8) |
| Maximum thickness of the effusion | 27 (24.1) |
| The use of calculation formulas | 5 (4.5) |
| Other methods | 13 (11.6) |
| Diaphragm assessment, n (%) | 14 (12.5) |
| Diaphragmatic excursion assessment, n (%) | 14 (12.5) |
| Diaphragmatic thickness, n (%) | 8 (7.1) |
| Length of the diaphragmatic zone of apposition, n (%) | 3 (2.7) |
| Clinical indication for diaphragm assessment I: mechanically ventilated patients, n (%) | 4 (3.5) |
| Clinical indication for diaphragm assessment II: restrictive or neuromuscular diseases, n (%) | 7 (6.3) |
| Clinical indication for diaphragm assessment III: obstructive diseases and/or COPD, n (%) | 4 (3.5) |
| Clinical indication for diaphragm assessment IV: acute respiratory failure, n (%) | 9 (8.0) |
